# Methotrexate-associated lymphoproliferative disorders in the central nervous system and stomach: A case report

**DOI:** 10.1097/MD.0000000000019850

**Published:** 2020-04-10

**Authors:** Mai Kawazoe, Kaichi Kaneko, Toshihiro Nanki

**Affiliations:** Division of Rheumatology, Department of Internal Medicine, School of Medicine, Faculty of Medicine, Toho University, Ota-ku, Tokyo, Japan.

**Keywords:** central nervous system lymphoma, methotrexate, methotrexate-associated lymphoproliferative disorder, rheumatoid arthritis

## Abstract

**Rationale:**

Methotrexate-associated lymphoproliferative disorder (MTX-LPD) is a serious complication in patients treated using methotrexate. It occasionally develops in extra-nodal sites, but rarely in the central nervous system (CNS) or in 2 different sites at the same time. We present the rare case of a patient with rheumatoid arthritis who developed lymphoma in the CNS and stomach during MTX therapy.

**Patient concerns:**

A 75-year-old Japanese man with rheumatoid arthritis who received methotrexate was admitted to our hospital because of gait ataxia and anorexia.

**Diagnoses:**

Imaging findings and biopsy led to a diagnosis of 2 different types of MTX-LPD in the central nervous system and stomach.

**Interventions:**

The lesion in his stomach improved after methotrexate withdrawal, whereas the cerebellar mass required high-dose methotrexate and rituximab therapy.

**Outcomes:**

Complete remission has been maintained for the 2 years following the initiation of chemotherapy.

**Lessons:**

In patients with RA who receive MTX and develop new neurological symptoms, CNS lymphoma as an MTX-LPD may be considered as a differential diagnosis.

## 1 Introduction

Methotrexate (MTX) is an anchor drug in the management of rheumatoid arthritis (RA). Lymphoproliferative disorder (LPD) occasionally develops in patients treated using MTX, and is termed MTX-associated LPD (MTX-LPD). The predominant primary site of MTX-LPD is the lymph nodes, followed by extra-nodal sites such as the gastrointestinal tract, skin, and lungs.^[1]^ The pathophysiology of MTX-LPD is not well understood. The hyperimmune state of RA itself or the immunosuppressive state induced by MTX administration is thought to play a role in the development of LPD.

The increased risk of LPD in RA patients has been well established. The incidence rate of LPD is 2-times higher in Western RA patients and 6-times higher in Japanese RA patients than that in the general population.^2^,^3^ However, LPD in the central nervous system (CNS) is rare because it accounts for less than 1% of LPD.^[4]^ We present the rare case of an RA patient who developed lymphoma in the CNS and stomach during MTX therapy.

## 2 Case report

**Figure 1:**
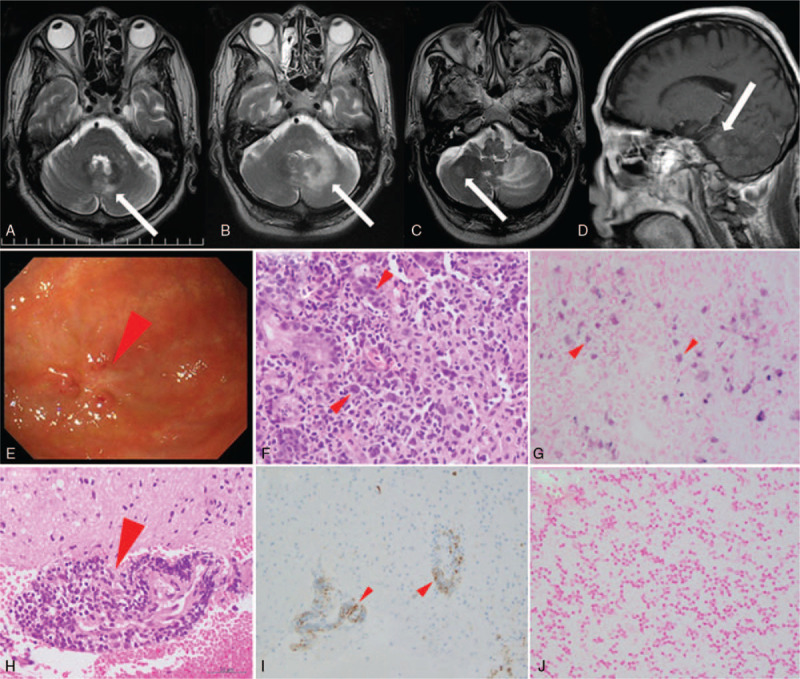
MTX-LPD in the CNS and stomach. T2-weighted MRI revealed a high-intensity area in the left cerebellum on the day of admission (A); an expanded high-intensity area on day 28 (B); new high-intensity areas in the right cerebellum (C) and pons (D) on day 40. Esophagogastroduodenoscopy revealed an ulcerative lesion in the greater curvature of the stomach (E). The histopathological finding was an ulcerative lesion. Hematoxylin and eosin staining showed the infiltration of atypical lymphocytes with irregular nuclear contours: ×400 (F). In situ hybridization confirmed Epstein–Barr virus-encoded ribonucleic acid in the nuclei of lymphoma cells: ×400 (G). Histological and immunohistochemical findings of the left cerebellar mass. Hematoxylin and eosin staining demonstrated the proliferation of large lymphocytes around the vessel: ×400 (H). Immunostaining for CD20 was positive in large cells: × 400 (I). In situ hybridization showed no Epstein–Barr virus-encoded ribonucleic acid-positive lymphoma cells: × 400 (J). CNS = central nervous system, MRI = magnetic resonance imaging, MTX = methotrexate, MTX-LPD = methotrexate-associated lymphoproliferative disorder.

The patient was a 75-year-old Japanese man with a 9-year history of seropositive RA. The disease activity of RA was controlled well by MTX at 14 mg/week and bucillamine at 100 mg/day. He was admitted to our hospital with a 2-day history of gait ataxia. He walked slightly to the left. Neurological examination revealed dysmetria and the deterioration of movement in the left upper limb. Hematological and biochemical tests demonstrated no abnormalities, except for a slight increase in the level of the serum-soluble interleukin-2 receptor (697 U/mL). Serological tests for the Epstein–Barr virus (EBV) antibody confirmed previous infection (EBV-VCA IgG × 160, EBV-VCA IgM negative, EBNA × 40). The cerebrospinal fluid protein content was high (72 mg/dL). The cell count and glucose and β2-microglobulin levels were normal. Cytology was normal. Magnetic resonance imaging (MRI) demonstrated a T2/FLAIR hyper-intense lesion in the left cerebellum (Fig. 1A). Diffusion-weighted imaging revealed high-intensity signals in the same area and apparent diffusion coefficient maps visualized the lesion with low intensity. No abnormalities on vascular imaging of the brain were observed on magnetic resonance angiography; thus, we suspected infarction and initiated aspirin treatment. However, nausea and anorexia developed, and gait ataxia persisted. Esophagogastroduodenoscopy revealed an ulcerative lesion in the greater curvature of the stomach (Fig. 1E). Biopsy demonstrated the proliferation of atypical lymphocytes with irregular nuclear contours in the gastric lamina propria (Fig. 1F). On immunohistochemical staining, atypical lymphocytes were positive for CD30, but not for CD20. In situ hybridization revealed EBV-encoded ribonucleic acid (EBER) in the nuclei of atypical lymphocytes (Fig. 1G). The gastric ulcerative lesion was diagnosed as gastric lymphoma, but esophagogastroduodenoscopy performed 2 weeks after withdrawal of MTX demonstrated that it was cured. However, anorexia persisted and gait ataxia gradually worsened. Moreover, an enlarged T2/FLAIR hyper-intense lesion in the left cerebellum became evident on MRI (Fig. 1B). Gadolinium-enhanced MRI demonstrated the non-enhancement of the lesion, we concluded this lesion to be cerebral infarction. Although aspirin was changed to clopidogrel bisulfate, the patient began to speak with long pauses between words, so-called scanning speech. MRI on the 40th day revealed new high-intensity areas in the right cerebellum and pons (Fig. 1C and D). Biopsy of the left cerebellar mass revealed the proliferation of atypical lymphocytes surrounding blood vessels (Fig. 1H). Immunohistochemical staining demonstrated positivity for CD20 (Fig. 1I), and the patient was diagnosed with diffuse large B-cell lymphoma (DLBCL). EBER was not detected (Fig. 1J). No other lesions were found on systemic computed tomography and bone marrow examination. As the lesion in the brain and that in the stomach were 2 different clones, the patient was diagnosed with primary central nervous system lymphoma (PCNSL). Based on his history of receiving MTX for RA, he was diagnosed with MTX-LPD. The clinical stage was IVA (cerebellum) and the national comprehensive cancer network-international prognostic index (NCCN-IPI) score was 6 (age 75 years old (2), serum lactate dehydrogenase (LDH) 194 U/L (1), CNS involvement (1), stage IV (1), performance state 4 (1)).

The brain lesion did not respond to withdrawal of MTX; therefore, chemotherapy with high-dose MTX (3.5 g/m^2^) and rituximab (375 mg/m^2^) was initiated. The mass lesion slowly regressed thereafter and resolved completely after 7 cycles of chemotherapy. His physical condition also improved. Complete remission has been maintained for the 2 years following the initiation of chemotherapy.

## 3 Discussion

We report the case of an RA patient who simultaneously developed 2 different types of LPD: gastric lymphoma with CD30 that was positive for EBER, but negative for CD20 and CNS lymphoma that was positive for CD20, but negative for EBER. To the best of our knowledge, only 5 cases of MTX-LPD that developed in the CNS have been reported to date (Table 1).^5^,^6^,^7^,^8^,^9^ The histology of LPD was DLBCL in most cases. The 4 patients who were able to be followed for LPD did not develop recurrence for more than 1 year after withdrawal of MTX, or after surgery or radiotherapy following the MTX cessation. Two patients, including ours, were treated by high-dose MTX. High-dose MTX therapy is the standard treatment for PCNSL, even if it is MTX-LPD, because it permeates the blood-brain barrier. Increased rates of complete remission^[10]^ and survival^[11]^ have been reported following the introduction of rituximab for PCNSL. This patient had an NCCN-IPI score of 6 and was included in the high-risk group. Considering that EBV-negative DLBCL, which does not regress after MTX cessation, has a poor prognosis,^[12]^ and chemotherapy is often required in EBV-negative cases,^[13]^ we chose a combination of high-dose MTX and rituximab therapy, resulting in complete remission. The lymphoma has not recurred for more than 2 years.

**Table 1:**
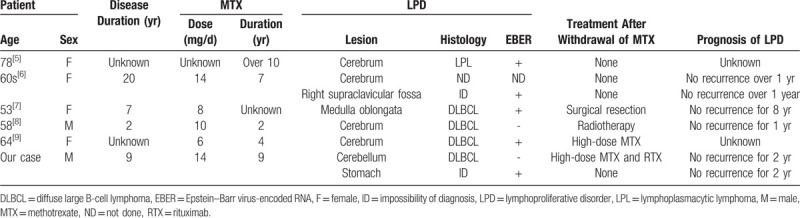
Summary of 6 patients who developed MTX-LPD in the CNS.

Hoshida et al^[1]^ previously reported that the percentage of EBV positivity in patients with RA who developed LPD is 27.6%, which is 3-fold higher than that in those with sporadic LPD. However, no significant differences were observed in the positive rate of EBV between RA patients treated with or without MTX. The characteristic of MTX-LPD is spontaneous regression after the withdrawal of MTX. By reviewing patients achieving complete remission of MTX-LPD, spontaneous remission mostly occurred within 4 weeks of the discontinuation of MTX.^[14]^ Ichikawa et al^[15]^ reported that regression was associated with EBV positivity. However, the spontaneous regression rate of MTX-LPD after only withdrawal of MTX was 22% to 59%, with the remaining patients requiring chemotherapy.^1^,^15^ In the present case, lymphoma cells were positive for EBER in the stomach, but were negative in the CNS. This may be why CNS lymphoma required chemotherapy, whereas gastric lymphoma regressed after the discontinuation of MTX.

## 4 Conclusion

We report the rare case of an RA patient who simultaneously developed lymphoma in the CNS and stomach during MTX therapy. Now that MTX has become commonly used, MTX-LPD cases have increased. Thus, in patients with RA who are treated using MTX and develop new neurological symptoms, CNS lymphoma as an MTX-LPD may be considered as a differential diagnosis.

## Author contributions

MK was a major contributor in writing the manuscript. All authors read and approved the final manuscript.

**Supervision:** Kaichi Kaneko, Toshihiro Nanki.

**Writing – original draft:** Mai Kawazoe.

**Writing – review & editing:** Mai Kawazoe, Toshihiro Nanki.

Toshihiro Nanki orcid: 0000-0002-3482-074X.
